# Soft-Palate Changes After Orthognathic Surgery: A Three-Dimensional Assessment of Positioning and Morphology

**DOI:** 10.3390/jpm16030141

**Published:** 2026-03-02

**Authors:** Orion Luiz Haas Junior, René de Jesús Quiñones Ravelo, Rubens Martins Bastos, Bibiana Mello da Rosa, Rogério Belle de Oliveira, Pedro Gomes de Oliveira, Robson Capasso

**Affiliations:** 1 Department of Sleep Surgery, Otolaryngology, Head and Neck Surgery, Stanford University, Stanford, CA 94305, USA; 2 Belle & Haas Orthofacial Surgery Centre, Porto Alegre 90440-060, RS, Brazildr.rogeriobelle@hotmail.com (R.B.d.O.); 3 Oral and Maxillofacial Surgery, Hospital São Lucas, Porto Alegre 90610-000, RS, Brazil; 4 Department of Oral and Maxillofacial Surgery Resident, Centro Médico Nacional 20 de Noviembre, ISSSTE, Ciudad de México 03229, Mexico; reneaobjj@gmail.com

**Keywords:** orthognathic surgery, airway remodeling, pharynx, soft palate, Le Fort osteotomy, diagnostics, personalized medicine

## Abstract

**Background/Objectives**: This study evaluated, by cone-beam computed tomography, the role of soft-palate morphology and positioning in upper airway volume and minimum cross-sectional area (mCSA) after orthognathic surgery at three time points: one week before surgery (T0); 4–6 months after surgery (T1); and 12–36 months after surgery (T2). **Methods**: Patients (N = 91) were divided into five groups according to maxillary surgical movement: 1: maxillary advancement; 2: maxillary advancement and counterclockwise rotation with anterior upward movement; 3: maxillary advancement and counterclockwise rotation with posterior downward movement; 4: maxillary advancement and clockwise rotation with anterior downward movement; and 5: maxillary advancement and clockwise rotation with posterior upward movement. **Results**: Time was an important predictor of change for almost all volume and mCSA parameters (*p* < 0.05), except for mCSA-nasopharynx (*p* = 0.114). Groups 1 and 5 showed recurrence of oropharynx volume and minimum cross-sectional area at 12–36 months, while Group 3 had 85% of vertical soft palate morphology and no oblique morphology at 12–36 months. **Conclusions**: Soft palate angulation did not change at any time or with any type of surgical movement. Maxillary counterclockwise rotation with posterior downward movement seems to be the preferred surgical movement of the maxilla to increase pharyngeal dimensions and improve soft-palate morphology.

## 1. Introduction

Orthognathic surgery is a safe and predictable procedure for the correction of dentofacial deformities. Its main indications are cosmesis, impaired masticatory function, and sleep-related breathing disorders [1]. Three-dimensional (3D) repositioning of the jaws alters the positions of soft tissues and generates volumetric changes in the upper airway. This occurs because the walls of the pharyngeal airway expand or contract depending on the type, direction, and extent of the facial skeletal movement [2,3].

Contemporary maxillomandibular advancement (MMA) for obstructive sleep apnea (OSA) is in a transitional moment from a “standardized surgical movement” paradigm to a patient-specific, diagnosis-driven strategy. Historically, many surgical algorithms—including those derived from the Stanford experience—have emphasized substantial skeletal advancement (often ≥10 mm) to maximize multilevel airway enlargement; however, more recent literature underscores that the magnitude and vector of movement should be individualized to the patient’s anatomical and pathophysiologic phenotype rather than applied as a uniform threshold [4]. This shift has been enabled by multimodal preoperative assessment integrating clinical evaluation, craniofacial and upper-airway imaging, and sleep-specific testing, which together support precision selection of surgical objectives and anticipated sites of collapse [5,6].

In parallel, digital workflows—virtual surgical planning (VSP—have facilitated accurate execution of individualized movement plans while permitting nuanced adjustments that balance airway goals with facial harmony and occlusal stability. Three-dimensional airway analyses after CAD/CAM-assisted MMA further reinforce the relevance of tailoring movements to patient-specific anatomy, supporting a precision approach in which surgical vectors are customized according to multimodal diagnostic findings rather than predetermined “one-size-fits-all” advancements [3,7,8].

While MMA and mandibular muscle repercussions have been explored in several studies, the specific impacts of such orthognathic surgery on the positioning and morphology of the soft palate remain unclear [3,9,10,11,12,13]. The soft palate is a 3D muscular structure located posterior to the hard palate. Together with the posterior and lateral pharyngeal walls, it forms a muscular valve known as the velopharyngeal sphincter [2]. Improvements in the upper airway due to surgical movements in the retropalatal regions are secondary to changes in the attachment of the soft palate to the posterior maxilla [11].

Within this context, this study aimed to evaluate the role of soft palate morphology and positioning in upper airway volume and constricted areas after orthognathic surgery, using 3D cone-beam computed tomography (CBCT) and voxel-based superimposition.

## 2. Materials and Methods

A longitudinal study design was employed, enrolling consecutive patients who presented from 2020 to 2024 at the Belle and Haas Orthofacial Surgery Centre/São Lucas Hospital (Porto Alegre, Brazil). All patients were included and operated on between 2021 and 2024. For some patients, preoperative cone-beam computed tomography (CBCT) scans obtained in 2020 were available and were used for diagnostic assessment, virtual surgical planning, and baseline preoperative evaluation (T0). The project was approved by the local Research Ethics Committee (46121421.2.0000.5336), and all study procedures were compliant with the Declaration of Helsinki.

The inclusion criteria were as follows: healthy, body mass index < 30, non-growing, non-syndromic patients with dentofacial deformities requiring orthognathic surgery (specifically, MMA), no history of previous facial trauma or surgery, and written informed consent for participation. Patients were excluded from this study if they had any systemic diseases, congenital anomalies, or incomplete postoperative follow-up.

All patients underwent MMA, performed by the same surgeons/authors (OLHJ and RBO). Patients were categorized into 5 groups by maxillary surgical movement: Group 1, patients who underwent maxillary advancement alone; Group 2, those who underwent maxillary advancement and counterclockwise rotation with anterior upward movement of the maxilla; Group 3, those who underwent maxillary advancement and counterclockwise rotation with posterior downward movement of the maxilla; Group 4, those who underwent maxillary advancement and clockwise rotation with anterior downward movement of the maxilla; and Group 5, those who underwent maxillary advancement and clockwise rotation with posterior upward movement.

Patients were evaluated by CBCT at three time points: 1 week before surgery (T0), 4–6 months after surgery (T1), and 12–36 months after surgery (T2).

### 2.1. Operative Protocol

All patients underwent bimaxillary orthognathic surgery under general anesthesia and nasotracheal intubation. Surgical movements were planned according to patient-specific functional needs (bimaxillary advancement) and esthetic requirements (rotational adjustments), in accordance with our previously published philosophy for dentofacial surgical correction [14,15].

In all groups, the maxilla was approached through a minimally invasive approach. In brief, a maxillary incision was made in the buccal sulcus of the premaxilla from right lateral incisor to left lateral incisor, and a subspinal osteotomy was performed (keeping the nasal muscles and septum attached to the anterior nasal spine), followed by Le Fort I osteotomy and pterygomaxillary disjunction using the “twist technique”. All osteotomies were stabilized using miniplates and screws. Finally, alar cinch sutures and V-Y closure were performed [16].

Postoperatively, patients received cold therapy with a cooling mask at 17 °C while in hospital admission and were discharged 24 h after surgery. Standard antibiotic and anti-inflammatory medications were prescribed.

### 2.2. Study Variables

The variables of interest were demographic (patient age, type of dentofacial deformity) and those concerning the impact of orthognathic surgery on the upper airways and soft palate: minimum cross-sectional area (mCSA)–nasopharynx, mCSA—oropharynx, and mCSA—pharynx relative to soft palate, nasopharynx volume, oropharynx volume, volume of pharynx relative to soft palate, soft palate morphology, and soft palate angulation.

### 2.3. CBCT Evaluation

The CBCT dataset was collected from i-CAT (Imaging Sciences International, Hatfield, PA, EUA; 120 kV, 8 mA, voxel size 0.3 mm) in Digital Imaging and Communications in Medicine (DICOM) format and processed using Dolphin 3D Orthognathic Surgery Planning Software version 11.9 (Dolphin Imaging and Management Solutions, Chartsworht, CA, USA), at three time points—T0, T1, and T2.

For CBCT acquisition, patients were instructed to sit upright in a natural head position, look forward, breathe calmly through the nose without swallowing, keep the tongue relaxed, and maintain the mandible in maximum intercuspation. The three CBCT datasets of each patient were superimposed in accordance with the voxel-based superimposition protocol [17].

### 2.4. Surgical Movements

**Figure 1:**
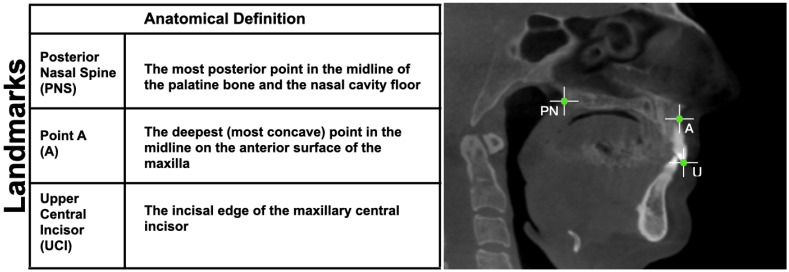
Schematic illustration and anatomical definition of reference landmarks used for surgical movement assessment.

To assess the magnitude and type of surgical movement performed, the following landmarks were used: Posterior Nasal Spine (PNS), Upper Central Incisor (UCI), and the most concave point of the anterior maxilla (Point A) (Figure 1).

### 2.5. Airway Analysis

**Figure 2:**
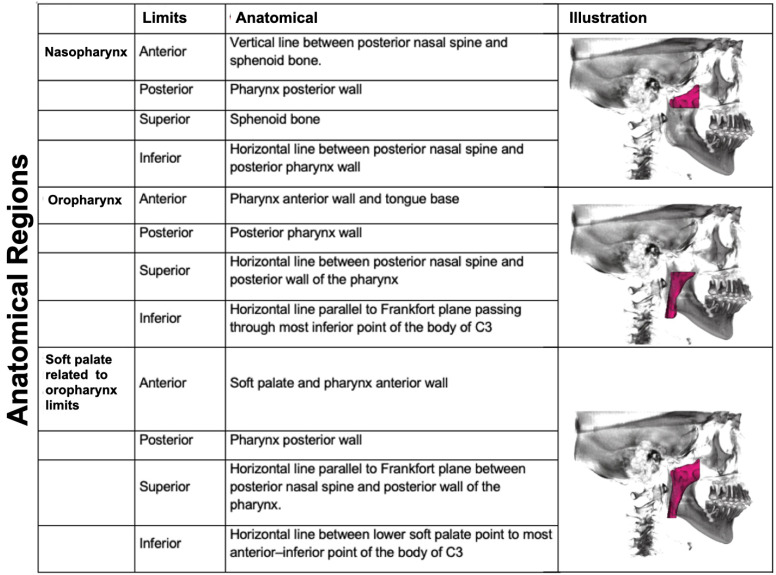
Three-dimensional anatomical structural segmentation of the airway, according to Swennen and Guijarro-Martínez [10].

Manual and 3D segmentation were performed to delimit the anatomical structures of the upper airway in all views as described by Swennen and Guijarro-Martínez [18]. The airway boundaries used in this study are described in Figure 2.

Volumetric airway changes (mm^3^) and maximum constricted-section area (mm^2^) were analyzed from the midsagittal plane. The airway was marked with a seed point tool, and the software automatically generated a full-color 3D image with numerical values.

### 2.6. Soft Palate Analysis

**Figure 3:**
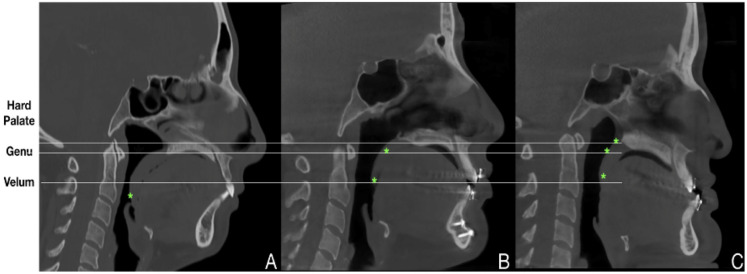
Patterns of soft palate morphology. (**A**). Oblique pattern at the velum. (**B**). Intermediate pattern at the velum and genu. (**C**). Vertical patterns at the velum, genu, and hard palate.

Two measurements were conducted to analyze soft palate morphology and angulation. For morphology, three anatomic landmarks were assessed on sagittal CBCT slices to verify soft palate patterns, as described by Woodson [11]: hard palate, genu, and velum (Figure 3). Interobserver agreement was assessed using kappa analysis between two independent authors (RJQR and OLHJ) to ensure measurement reliability.

For angulation assessment, the angle formed by the intersection of the lower soft palate (LSP), posterior nasal spine (PNS), and Point A was measured to evaluate whether the soft palate musculature followed the skeletal movements after surgery.

### 2.7. Statistical Analysis

Data were analyzed in the SPSS v20.0 software environment. The normality of variables was assessed by the Kolmogorov–Smirnov test. Between-group comparisons were performed using the chi-square (x^2^) test for categorical variables and analysis of variance (ANOVA) for quantitative variables with normal distribution. When significant associations were detected, proportions were compared using a Bonferroni adjustment. For variables with asymmetric distribution, the Kruskal–Wallis test was used instead, followed by the Dunn–Bonferroni procedure for multiple comparisons.

Finally, comparison of categorical variables between time points was performed using the McNemar test. Generalized estimating equation (GEE) analysis followed by the Bonferroni test was used to compare measurements over time between groups. Statistical significance was set at 5%.

The intraclass correlation coefficient (ICC) was evaluated by two authors (RJQR and OLHJ).

## 3. Results

A total of 91 adult patients who had undergone MMA were enrolled in this study (Group 1, n = 17, Group 2, n = 29; Group 3, n = 26; Group 4, n = 13; Group 5, n = 6). There were 63 females (69%) and 28 males (31%), with a mean age of 34 years (range, 24–60 years). Mandibular advancement between 5 and 10 mm. Regarding surgical movements of the maxilla, the average maxillary advancement was 3 mm, with a minimum advancement of 1.35 mm for Group 3 and a maximum of 4.8 mm for Group 2 (Table 1).

### 3.1. Airway Analysis

The mCSA-nasopharynx measurement did not show any interaction effect (neither time nor group). However, recurrence was observed in Groups 1 and 5 between T1 and T2. The other volumes and mCSA were significantly different, with an increase in measurements between T0 and T1 and T2 in all five groups. The results showed that the total airway size increased significantly from T0 to T2.

The mCSA-soft palate relative to oropharynx measurement and total volume exhibited a significant effect over time (*p* < 0.001), with an increase between T0 and T2 in all five groups.

Measurements of nasopharynx volume showed a significant effect of interaction (*p* < 0.001) and time (*p* < 0.001). At T1, there was a statistically significant difference between the means of Groups 1 and 3 (*p* = 0.022).

When comparing means within the groups, Group 2 exhibited a difference between T0 and T2 (*p* = 0.019); Group 3, between T0 versus T1 and T2 (*p* = 0.001 and *p* < 0.001 respectively); Group 4, between T0 and T1 in relation to T2 (*p* = 0.027 and *p* = 0.013 respectively); and Group 5, between T0 and T1 (*p* = 0.004) (Table 2).

### 3.2. Soft Palate Analysis

**Figure 4:**
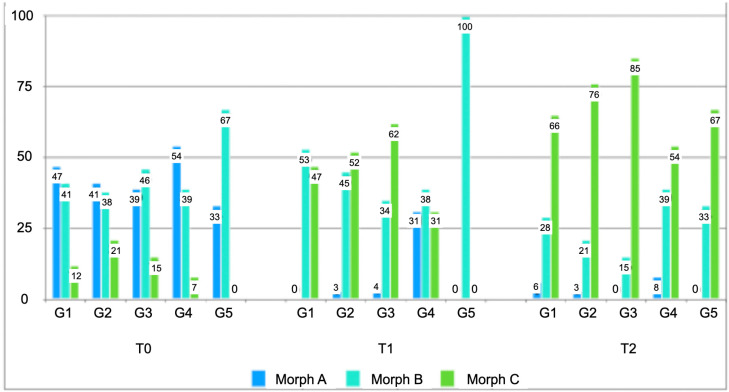
**Percentage distribution of soft palate morphology patterns among subgroups across three assessment time points.** T0 preoperative, T1 4–6 months postoperative, and T2 after 12 months postoperative. Morph A (Blue): Oblique soft-palate; Morph B (Light Blue): Intermediate soft-palate; Morph C (Green): Vertical soft-palate.

There was no effect of time, group, or time × group interaction on soft palate angulation. However, regarding soft palate morphology, there were significant differences (*p* = 0.003) between Groups 3 and 5 at T1. In Group 2, there was a significant difference in morphology from T0 to T1 (*p* = 0.003) and from T1 to T2 (*p* = 0.038). Group 3 showed significant improvements in morphology from T0 to T1 (*p* < 0.001); we could not evaluate the difference between T1 and T2, because there is no patient with morphology A in T2. Group 4 showed no significant difference from T0 to T1 (*p* = 0.343) nor from T1 to T2 (*p* = 0.469). Recurrence in soft-palate morphology was present between times T1 and T2 in Groups 1 and 5 (Figure 4).

Interobserver agreement was perfect for soft-palate morphology (κ = 1.00), and intraclass correlation coefficients (ICCs) demonstrated excellent reliability across all variables, ranging from 0.889 for surgical movements to 0.984 for airway analysis.

## 4. Discussion

The type of surgical movement imparted to the maxilla has a significant impact on personalized orthognathic surgery and should be considered carefully during surgical planning, particularly regarding the posterior portion of the maxilla and its relationship with the soft palate.

In our sample, MMA with counterclockwise rotation was the surgical movement that most increased volume and minimum cross-sectional area (constricted area), which corroborates previous studies [3,19,20,21,22,23]. Notably, there are several innovative findings to report. First, we were able to demonstrate that counterclockwise rotation outperforms anteroposterior advancement alone (without rotation) and clockwise rotation in terms of soft palate morphology, unlike Lee et al. [24], who suggested that maxillary posterior impaction and anterior movement at the posterior nasal spine tended to elevate the soft palate and constrict the velopharynx.

Considering these findings, the posterior maxilla is extremely important when studying soft-palate morphology because the muscular aponeurosis is attached exactly to that anatomic region; therefore, surgical movements have a direct influence on soft palate position and counterclockwise rotation with posterior downward placement of the maxilla seems to be the best surgical movement when the goal is to increase volume and minimum cross-sectional area [3,25] and change morphology to a vertical position.

Many studies have described the relationship of skeletal anatomy and soft-palate morphology with the upper oropharynx [11,26,27]. Class II patients have been associated with a more obliquely oriented airway and reduced oropharyngeal dimensions; patients with class III morphology have the largest oropharynx dimensions and have been associated with a more vertically oriented soft palate [26,27]. In our study, in Group 2, there was a significant difference in morphology from T0 to T1 (*p* = 0.003) and from T1 to T2 (*p* = 0.038). However, Group 3 showed significant changes in morphology between T0 and T1 (*p* < 0.001) and T1 and T2, showing no oblique morphology in T2, only intermediate and vertical.

In Group 3, 85% of patients had the soft palate in a vertical position and 15% in an intermediate position; this was the group in which the soft palate was best repositioned after surgery and throughout the follow-up period. This finding is consistent with the fact that the combination of maxillary advancement and counterclockwise rotation with posterior downward movement of the maxilla enlarges the pharynx because the muscles of the soft palate are pulled to an anterior and downward position, which favors enlargement of the upper airway space.

Our results did not show any modification of the palatal angle in relation to time, interaction, or group, which suggests the surgical movements were directly reproduced from bone to the palatal muscular aponeurosis, showing significant increases in volume and minimum cross-sectional area in the immediate postoperative period (T1), which coincides with previous studies [3,23,27]. This also corroborates our findings that counterclockwise rotation with posterior downward maxillary movement repositioned the soft palate into a more vertical position, opening the pharynx.

Consistent with the biological plausibility concerning the attachment of the soft palate muscles in the posterior maxilla, this study also showed recurrence of volume, mCSA, and soft palate morphology from T1 to T2 in Groups 1 and 5; as, there were no BMI changes during the follow-up, the findings coincide with Giralt-Hernando et al. [3] where maxillary advancement without rotation yields enlargement of the upper airway at first, followed after some months by a particular form of recurrence known as “elastic effect”. The effect in Group 5 was the same as in Group 1; the initial muscle response is advancement and tension, followed by recurrence after a few months because the posterior maxilla was brought to a more restricted area of the airway, closer to the upper pharyngeal walls. The levator veli palatini muscle comprises 40% of the soft palate length between the base of the uvula and the hard palate. The orientation, size, length, and vectors of the palatal muscles are major determinants of the palatal airway [26].

Another interesting finding is that, despite Group 3 exhibiting the least maxillary advancement, with an average ranging from 1.35 to 3.12 mm, it did not show any specific recurrence and achieved results equal to or better than those of the other groups, especially when compared to Group 2, which also underwent counterclockwise rotation, but had an average maxillary advancement ranging from 2.70 to 4.80 mm. The anterior position of the rotation fulcrum (in Group 3, the anterior nasal spine) accentuates the displacement of the palate in an inferior-anterior direction, increasing dimensions at all levels of the pharyngeal airway [3,23,28].

The facial repercussions of linear maxillary advancement can compromise the desired outcome when considering the treatment of patients with aesthetic concerns, in addition to respiratory factors. The described findings represent a major improvement, as they make it possible for the surgeon to avoid over-projected faces, thereby balancing both aesthetic and functional effects.

Some limitations regarding the study population must be acknowledged. Although patients with BMI < 30 were excluded, the sample was not stratified according to BMI subgroups, which could potentially influence changes in volume and minimum cross-sectional area (mCSA) over time. Nevertheless, BMI does not modify the position or inclination of the soft palate. Therefore, considering that the groups presenting the highest rates of recurrence were also those with a greater proportion of individuals exhibiting soft palate morphology type A, it is reasonable to infer that the recurrence was primarily attributable to the surgical movement itself rather than to BMI increase. Second, although the present analysis was not conducted in a cohort diagnosed with obstructive sleep apnea (OSA)—thereby limiting our ability to determine the full physiologic repercussions of maxillary surgical movements in a pathologic, sleep-disordered population—the findings nonetheless offer a valuable indication of how palatal morphology responds to surgical manipulation. Such insights contribute meaningfully to the refinement of patient-specific planning, enabling a more precise personalization of maxillary surgical movements according to the anatomical and functional requirements of each individual.

**Figure 5:**
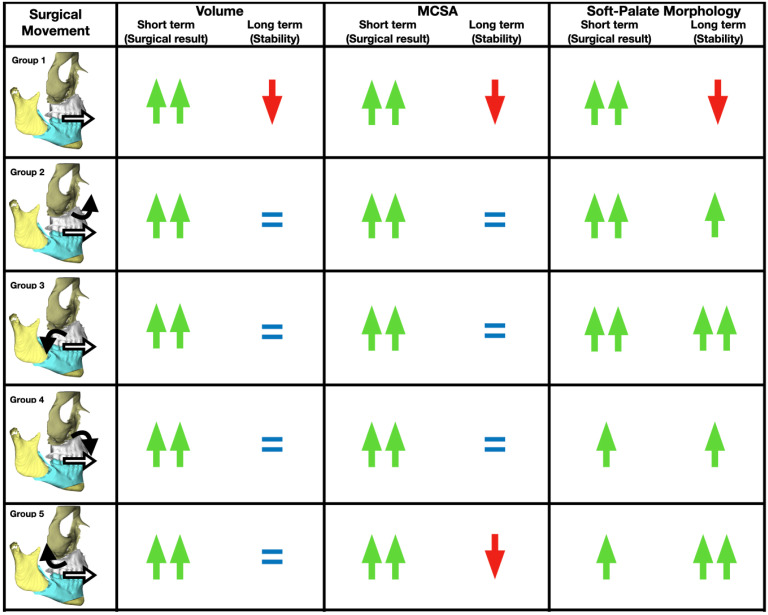
Schematic overview of study groups and corresponding outcomes for clinical correlation. Green arrows denote increased values, horizontal bars denote no change, and red arrows denote decreased values.

In conclusion, counterclockwise rotation with posterior downward movement of the maxilla demonstrated the most significant gain in volume and minimum cross-sectional area, as well as vertical morphological changes in the airway (Figure 5).

The results of this study suggest that, whenever the case allows, preference should be given to rotation rather than linear maxillary movements to achieve better functional and aesthetic outcomes in patients undergoing MMA.

## Figures and Tables

**Table 1:** Comparison of surgical skeletal displacements between intervention groups from T0 to T1.

	Group 1 n = 17			Group 2 n = 29			Group 3 n = 26			Group 4 n = 13			Group 5 n = 6			*p*
	Median	Percentile		Median	Percentile		Median	Percentile		Median	Percentile		Median	Percentile		
		25	75		25	75		25	75		25	75		25	75	
**Transversal**																
**PNS**	0.00	−0.10	0.20	0.00	−0.85	0.15	0.00	−0.73	0.60	0.10	0.00	1.70	0.00	−1.00	0.10	0.207
**A point**	0.00	−0.25	0.20	0.00	−0.85	0.40	0.00	−0.80	0.60	0.20	−0.10	1.70	0.00	−0.23	0.18	0.417
**UCI**	0.00	−0.55	0.40	0.00	−0.95	0.60	0.00	−1.03	0.20	0.40	0.05	1.70	0.00	−0.23	0.18	0.065
**Vertical**																
**PNS**	0.10	−0.05	0.60	0.40	−0.80	1.60	3.20	2.15	3.80	0.30	−0.30	1.10	−3.20	−4.93	−1.38	<0.001
**A point**	0.20	−0.20	0.45	−3.10	−4.30	−1.70	0.10	−0.08	0.78	1.60	0.90	2.80	−0.05	−2.88	1.40	<0.001
**UCI**	0.20	−0.40	0.40	−3.80	−5.75	−2.70	−0.60	−1.50	0.63	3.20	1.35	4.10	−0.05	−1.88	0.68	<0.001
**Sagittal**																
**PNS**	2.80	1.65	3.50	2.70	2.30	4.05	1.65	0.20	3.85	3.00	0.65	4.95	4.05	3.15	6.03	0.065
**A point**	2.30	1.80	3.70	3.50	2.70	5.05	1.35	0.55	2.63	2.60	1.50	3.90	2.50	1.93	3.68	<0.001
**UCI**	2.80	2.00	4.25	4.80	3.75	6.20	3.15	2.23	4.50	2.70	0.75	4.75	3.00	2.33	4.03	0.001

Data compared by the Kruskal–Wallis test followed by the Dunn–Bonferroni post hoc test. Group 1: Linear maxillary advancement; Group 2: Maxillary advancement + CCWR + anterior upward; Group 3: Maxillary advancement + CCRW + posterior downward; Group 4: Maxillary advancement + CWR + anterior downward; Group 5: Maxillary advancement + CWR + posterior upward. PNS: posterior nasal spine. A point: anterior maxilla. UCI: upper central incisor.

**Table 2:** Longitudinal comparison of measurements between groups.

	Group 1 n = 17		Group 2 n = 29		Group 3 n = 26		Group 4 n = 13		Group 5 n = 6		*p* Interaction	*p* Time	*p* Group
	Average	SD	Average	SD	Average	SD	Average	SD	Average	SD			
**MCSA** **oropharynx**											0.664	<0.001	0.493
T0	173.8	111.9	170.4	96.1	184.5	103.4	181.2	115.1	147.3	121.0			
T1	219.8	127.2	239.1	126.9	243.7	115.2	199.2	178.1	165.7	83.3			
T2	197.5	123.1	246.2	104.3	242.4	101.3	264.4	202.2	173.0	108.4			
**MCSA** **nasopharynx**											0.234	0.114	0.566
T0	210.7	137.1	246.1	120.9	225.7	120.7	284.8	177.1	254.7	136.1			
T1	256.4	165.4	228.2	138.8	287.4	164.8	272.1	170.7	279.0	154.6			
T2	238.1	138.8	283.3	168.4	338.3	154.1	331.8	188.7	205.5	133.1			
**MCSA** **pharynx/soft palate**											0.075	<0.001	0.235
T0	190.4	114.4	192.0	105.8	222.2	114.8	205.2	151.0	186.3	132.3			
T1	229.5	167.9	271.3	131.5	294.6	138.7	216.0	188.8	230.8	152.0			
T2	202.1	130.8	293.6	136.6	309.4	133.4	327.8	208.5	226.7	91.0			
**Nasopharynx** **volume**											<0.001	<0.001	0.061
T0	7536.1	1755.9	7926.1	2281.1	8074.2	1939.6	8046.3	2605.7	8765.8	2502.1			
T1	8005.4	1650.0	8527.5	2134.3	10,373.4	3468.6	7654.5	3146.2	7501.8	2623.9			
T2	8377.9	2083.9	9055.2	3032.7	9952.6	2333.4	10,195.8	4564.6	8513.2	2084.4			
**Oropharynx** **volume**											0.431	0.001	0.460
T0	12,969.7	4551.8	14,058.5	5977.7	14,234.8	5696.6	11,592.0	5511.4	11,979.3	6578.0			
T1	16,059.0	6502.3	16,440.0	6745.8	15,238.7	6529.0	12,547.9	7808.9	13,686.8	3777.9			
T2	15,175.2	7694.7	17,442.9	6799.4	15,020.5	6131.9	16,133.7	9113.5	15,284.5	1478.7			
**Pharynx** **Soft palate volume**											0.225	0.004	0.391
T0	9080.3	4534.1	8777.4	3824.4	9630.6	4268.9	8724.5	4901.3	9807.0	2613.0			
T1	10,539.6	4351.3	10,505.8	4423.8	11,259.1	4350.5	8270.0	5502.7	9502.0	3086.9			
T2	9282.8	5130.9	11,483.1	4552.7	12,768.3	6645.1	11,447.8	7160.2	10,206.2	1055.0			
**Soft palate** **angulation**											0.549	0.176	0.114
T0	119.0	8.1	124.7	26.8	114.9	10.2	130.6	34.2	121.0	13.1			
T1	118.9	7.5	120.4	10.4	115.5	8.9	119.2	7.7	118.6	11.5			
T2	122.0	17.4	118.9	10.2	117.2	8.9	120.7	7.0	120.9	6.9			

SD: standard deviation. *p*-values obtained by the Generalized Estimating Equations Model. Group 1: Linear maxillary advancement; Group 2: Maxillary advancement + CCWR + anterior upward; Group 3: Maxillary advancement + CCWR + posterior downward; Group 4: Maxillary advancement + CWR + anterior downward; Group 5: Maxillary advancement + CWR + posterior upward.

## Data Availability

The data presented in this study are available on request from the corresponding author. The data are not publicly available due to ethical and privacy restrictions.
